# cGMP-Phosphodiesterase Inhibition Prevents Hypoxia-Induced Cell Death Activation in Porcine Retinal Explants

**DOI:** 10.1371/journal.pone.0166717

**Published:** 2016-11-18

**Authors:** Lorena Olivares-González, Cristina Martínez-Fernández de la Cámara, David Hervás, María Pilar Marín, Agustin Lahoz, José María Millán, Regina Rodrigo

**Affiliations:** 1 Grupo de Biomedicina Molecular, Celular y Genómica, Instituto de Investigación Sanitaria La Fe, Valencia, Spain; 2 CIBER de Enfermedades Raras (CIBERER), Madrid, Spain; 3 Unidad de Bioestadística, Instituto de Investigación Sanitaria La Fe, Valencia, Spain; 4 Unidad de Microscopía, Instituto de Investigación Sanitaria La Fe, Valencia, Spain; 5 Unidad de Hepatología Experimental, Unidad Analítica, Instituto de Investigación Sanitaria La Fe, Valencia, Spain; University of PECS Medical School, HUNGARY

## Abstract

Retinal hypoxia and oxidative stress are involved in several retinal degenerations including diabetic retinopathy, glaucoma, central retinal artery occlusion, or retinopathy of prematurity. The second messenger cyclic guanosine monophosphate (cGMP) has been reported to be protective for neuronal cells under several pathological conditions including ischemia/hypoxia. The purpose of this study was to evaluate whether the accumulation of cGMP through the pharmacological inhibition of phosphodiesterase (PDE) with Zaprinast prevented retinal degeneration induced by mild hypoxia in cultures of porcine retina. Exposure to mild hypoxia (5% O_2_) for 24h reduced cGMP content and induced retinal degeneration by caspase dependent and independent (PARP activation) mechanisms. Hypoxia also produced a redox imbalance reducing antioxidant response (superoxide dismutase and catalase activities) and increasing superoxide free radical release. Zaprinast reduced mild hypoxia-induced cell death through inhibition of *caspase-3* or PARP activation depending on the cell layer. PDE inhibition also ameliorated the effects of mild hypoxia on antioxidant response and the release of superoxide radical in the photoreceptor layer. The use of a PKG inhibitor, KT5823, suggested that cGMP-PKG pathway is involved in cell survival and antioxidant response. The inhibition of PDE, therefore, could be useful for reducing retinal degeneration under hypoxic/ischemic conditions.

## Introduction

Retinal cell death derived from ischemia occurs in several retinal diseases including central retinal artery occlusion, glaucoma, diabetic retinopathy, retinopathy of prematurity, age-related macular degeneration, and ischemic central retinal vein thrombosis [1–5]. During retinal ischemia blood supply is reduced to an insufficient level leading to a lack of oxygen or hypoxia. This hypoxia can lead to serious consequences such as failure of energy balance causing ATP depletion, reactive oxygen species (ROS)-induced damage of cellular components, uncontrolled excitatory neurotransmitter release, inflammation and stimulation of the immune system [6], and neuronal and epithelial cell death [7, 8] or glial cells dysfunction [9, 10] in the retina. In general the inner retina layers are better protected from ischemic stress than other parts of the central nervous system; these cells are capable of recovering after an acute hypoxic insult. However, chronic retinal ischemia and hypoxia can lead to cell death and irreversible visual impairment [11–14].

Caspase-dependent [15–17] and–independent mechanisms of cell death as poly (ADP-ribose) polymerase (PARP) activation[18, 19] have been proposed during hypoxic situations in the retina. PARP activation is induced by reactive oxygen species (ROS) that produce nuclear DNA oxidative breaks [20]. This enzyme regulates multiple pathophysiological cellular processes including caspase-independent cell death through the formation of poly (ADP-ribose) polymers (PAR), which triggers nuclear translocation of apoptosis-inducing factor (AIF) and DNA condensation.

In ischemic/hypoxic retinopathies, hypoxia is accompanied by inflammation [21, 22] and the excess production of ROS that in turn, contribute to their pathogenesis [5, 23]. Both cellular processes are closely related. For instance, inflammation is exacerbated by further increases in ROS and reactive nitrogen species (RNS) production due to stimulation by cytokines (IL-6, TNFα) and growth factors [24–26].

The second messenger cyclic guanosine monophosphate (cGMP) is a cyclic derivative from the nucleotide guanosine triphosphate (GTP), which acts as second messenger in several cell pathways of signaling transduction such as phototransduction, muscular contraction, vasodilatation, platelet activation, sleep or memory among other functions [27]. It is generated by guanylyl cyclase (GC) which presents two isoforms, one soluble (sGC) and another solid or particulate (pGC). The sGC is activated by nitric oxide (NO), while the natriuretic peptide activates the pGC. Furthermore, the cGMP concentration is modulated by cGMP-degrading phosphodiesterases (PDEs) which hydrolyze it to 5’-GMP. cGMP employs several targets to exert its function. They comprise cGMP-dependent protein kinases (PKGI and PKGII), ion channels, and phosphodiesterases. In the retina, the cGMP performs an important role in the cascade of phototransduction which takes place in the photoreceptor (rods and cones) [28]. PDE1, PDE5 and PDE6 isoforms are found in mammalian retina [29] PDE5 and PDE6 share many structural, pharmacological and biochemical properties but differ in their cellular localization. While PDE6 is localized in photoreceptors, PDE5 is found in retinal and choroid vasculature, ganglion and bipolar cells [30].

The beneficial or deleterious role of the cGMP in the nervous system is controversial. Growing evidence supports a neuroprotective role for the NO-sGC-cGMP pathway in neuronal cells against apoptosis, especially for retinal cells [31]. For instance, NO inhibits apoptosis of retina neurons in culture through the cGMP/PKG pathway [32]. Under retinal ischemia, cGMP protects cells from cell death by inhibiting voltage dependent calcium channels and calcium influx [31]. Nipradilol, a nonselective beta and selective α_1_-adrenergic antagonist that can generate NO from a nitroxy residue, is capable of improving the survival rate of cultured retinal ganglion cells (RGCs) exposed to hypoxia *in vitro* [33] or ganglion cells from diabetic retinas [34]. On the other hand, cGMP or cAMP-degrading PDE inhibitors have been used as putative neuroprotective molecules in experimental models of retinal ischemia with positive results on retinal cells survival [35–37].

In the current study, we used porcine retinal explants exposed to mild hypoxia (5% O_2_) in the presence or the absence of the PDE5/6 inhibitor, Zaprinast, to investigate whether (1) hypoxia-induced cell death was prevented by PDE6 inhibition; (2) PDE6 inhibition prevented hypoxia-induced cell death through PKG activation; (3) hypoxia also affected antioxidant response and oxidative stress markers.

## Material and Methods

### Porcine retinal explant cultures

Sixty eyes (both left and right eyes from each animal) from pigs aged 3–7 months old were obtained from the local slaughterhouse Mercavalencia (Valencia, Spain). The eyes were collected from pigs that were sacrificed for commercial purposes, never for the experiments performed in this study. These tissues (the eyes of the pigs) would be discarded otherwise. Therefore this study did not need to be approved by an ethical committee. In any case animals were treated according to European Union standards and were sacrificed according to the current legislation (CE 1099/2009). Cultures of neuroretina explants were carried out as previously described [38]. Explants were cultured at 37°C under normoxia (21% of oxygen) or mild hypoxia conditions (5% of oxygen) for 24h. To evaluate the possible protective effect of cGMP accumulation, Zaprinast was used at 100 nmol/L as cGMP-specific phosphodiesterase inhibitor of PDE6 [38, 39]. Zaprinast can inhibit both PDE6 (IC50 = 150 nmol/L) and PDE5 (IC50 = 500–700 nmol/L), however, the inhibitory potency of Zaprinast for rod PDE6 and cone PDE6 (Ki = 30 nM and Ki = 32 nM, respectively) is four times higher than for PDE5 (Ki = 130 nM). As we chose a working concentration five-seven times lower than the IC50 for PDE, we believe that most of the cGMP accumulation is due to the inhibition of PDE6, in photoreceptors (rods and cones), rather than the inhibition of PDE5 in retinal vessels.

KT5823 was used at 1 μmol/L as potent and selective inhibitor of the PKG (IC50 = 234 nmol/L, Ki = 50–100 nM). KT5823 is also a weak inhibitor of PKC (Ki = 4 μmol/L) and PKA (Ki >10 μmol/L). The working concentration of KT5823 used in our study, 1 μM, is supposed not to affect PKA or PKC activity according to their Ki. Zaprinast (Sigma-Aldrich, Madrid, Spain) and KT5823 (AppliChem, Darmstadt, Germany) were diluted in dimethyl sulfoxide (DMSO). The equivalent amount of DMSO was added to the culture medium of controls. Treatments were added the day of the culture and maintained them for 24h.

### Tissue processing and histology

Retinal explants were fixed in 4% filtered paraformaldehyde (Sigma-Aldrich, Madrid, Spain) in 0.1 M PB (pH 7.4) and cryoprotected in a sucrose gradient (15-20-30%) (Panreac Química, Barcelona, Spain). Samples were frozen embedded in Tissue-Tek^®^ O.C.T.^™^ Compound (Sakura Finetek Europe B.V., Zoeterwoude, The Netherlands).Then, 10 μm sections were cut with a cryostat (Leica CM1900, Nussloch, Germany) and placed on Super Frost Ultra Plus treated slides (Thermo Scientific, Barcelona, Spain).

For immunofluorescence procedures, sections were post-fixed for 15 min at room temperature in 4% filtered paraformaldehyde (Sigma-Aldrich, Madrid, Spain) in 0.1 M PB (pH 7.4). Sections were incubated for one hour in blocking solution containing 5% normal goat serum, 1% bovine serum albumin and 0.25% Triton X-100. They were then incubated with primary antibody against PAR (1:200, Enzo Life Science, Madrid, Spain) as an indirect marker for PARP activity or against cleaved *caspase*-3 (1:200, Cell Signaling Technology, Barcelona, Spain) overnight at 4°C. Sections were then incubated with the fluorescence-conjugated secondary antibodies Alexa Fluor 488 or Alexa Fluor 647 (Invitrogen, Life Technologies, Madrid, Spain) for one hour at room temperature. After labelling and counterstaining with DAPI (Sigma-Aldrich, Madrid, Spain), the sections were mounted in Fluoromount-G (Southern Biotechnology, Birmingham, AL, USA) and observed at 22°C under a confocal microscope (40X magnification, Leica TCS SP5 Confocal microscope, Leica Microsystems CMS GmbH, Mannheim, Germany) belonging to the Microscopy Unit of the IIS-La Fe (Valencia, Spain). Leica LAS AF was used as microscope imaging software (Leica Microsystems CMS GmbH, Mannheim, Germany).

ImageJ software was used to quantify the images. To evaluate cell death, the terminal deoxynucleotidyl transferase dUTP nick and labelling (TUNEL) assay was used as previously described [38]. TUNEL-, PAR- and *caspase*-3 positive cells per visual field were counted in at least three visual fields per each retinal explant using software ImageJ software. The data were analysed quantitatively and, only cells with purple or green intensity were considered TUNEL-positive. The number of apoptotic nuclei was normalised to the SYTOX Green-labelled cell nuclei. Results are given as percentage of apoptotic nuclei/total nuclei. Data are expressed as mean ± SEM.

For biochemical determinations and gene expression analysis, retinal explants were placed immediately into the appropriate buffer and stored at -80°C.

### Lactate and pyruvate determination

40 mg of each retinal explant was homogenized in 3:10:27 chloroform/water/methanol mixture and centrifuge for 5 min at 10000 g at 4°C. Supernatants were used to determine lactate and pyruvate by the Gas Chromatography–Mass Spectrometry (GC-MS) analytical method (GC-Q-TOF system, consisting of an Agilent 7890 GC/7200 QTOF-MS, equipped with a capillary column coated with ZORBAX DB5-MS (30m X 250 μm X 0.25 μm) + 10m Duragard Capillary Column from Agilent). The mass spectral data were processed using MassHunter Qualitative Analysis B.07.

### Gene expression analysis

Total RNA was isolated from frozen retinal explants using RNeasy mini kit (Qiagen, Hilden, Germany) following the manufacturer’s protocol. Then, cDNA was synthesized starting from 1 μg of RNA by reverse transcription using the GeneAmp Gold RNA PCR Reagent kit (Applied Biosystems, Carlsbad, CA, USA) following manufacturer’s instructions. The relative gene expression of a subset of hypoxia inducible factor (HIF-1α) target genes: erythropoietin (EPO), vascular endothelial growth factor (VEGF), inducible nitric oxide synthase (iNOS) and adrenomedullin (ADM) were measured by real-time PCR using a commercial thermal-cycler (Applied Biosystems ViiATM 7 Real-Time PCR System; Life Technologies Corporation, Carlsbad, California, USA), TaqMan® gene expression assay (Ss03374608_u1 (iNOS), Ss03394058_g1 (ADM), Ss03382803_u1 (EPO), and Ss03393990_m1 (VEGF)) and TaqMan® 2X PCR Master Mix (Applied Biosystems, Life Technologies Corporation, Carlsbad, California, USA). Hypoxanthine phosphoribosyltransferase 1 (HPRT1) gene (Ss03388275_g1) was used as housekeeping gene.

Real-time PCR was performed with 1 cycle of 2 min at 50°C, followed by 1 cycle of denaturation of 10 min at 95°C, continued by 40 cycles of 15 seconds denaturation at 95°C and 60 seconds annealing at 60°C.

### Caspase-3 activity assay

*Caspase-3* activity was measured with a colorimetric tetrapeptide (DEVD-*p*NA) cleavage assay kit following the manufacturer's instructions (Bio-Vision, Mountain View, CA). Total retinal protein was extracted from retinal explants and measured by the BCA protein assay. *Caspase-3* activity was expressed as arbitrary units (au)/mg of protein.

### Cyclic GMP determination

cGMP was measured by using the BIOTRAK cGMP enzyme immunoassay kit (GE Healthcare Europe GmbH, Barcelona, Spain). Retinal explants were homogenized in 5% trichloroacetic acid and neutralized with 2M potassium bicarbonate. Neutralized supernatant was used for cGMP determination. Protein content was measured by the bicinchoninic acid (BCA) protein assay (BCA Kit; Pierce Scientific, CA). The tissue cGMP levels were expressed as pmol/mg protein.

### Soluble guanylate cyclase (sGC) activity assay

Retinal explants were homogenized in ice-cold buffer containing 50 mM HEPES (pH 7.4), 4 mM EDTA, 50% glycerol, 250 mM sucrose, and 1 mM dithiothreitol. To initiate the enzymatic reaction, samples were mixed rapidly with an equal volume of a buffer containing 50 mM HEPES (pH 7.4), 4 mM GTP, 60 mM phosphocreatine, 670 μg/ml creatine kinase (150U/mg), 1 mg/ml bovine serum albumin, and 8 mM MnCl_2_. Samples were incubated at 37°C for 5 min, boiled for 5 min and placed on ice for 10 min. After centrifugation at 12,000 x g for 10 min at 4°C, the supernatants were used to measure cGMP. sGC activity was expressed as pmol of cGMP/mg protein.min.

### Antioxidant response evaluation

Retinal explants were homogenized in 5 mM phosphate buffer pH 7, 0.9% NaCl, 0.1% glucose, centrifuged at 10,000 × g for 10 min at 4°C. Supernatants were used to determine total antioxidant capacity (TAC), cytosolic superoxide dismutase (SOD) and catalase (CAT) activities with commercial kits following Manufacturer´s Instructions (Cayman Chemical, Ann Arbor, MI, USA). Protein concentrations were measured by the BCA protein assay. Retinal TAC levels were expressed as nmol/mg protein. For total SOD activity assay one unit of SOD was defined as the amount of enzyme needed to exhibit 50% dismutation of the superoxide radical. SOD activity was expressed as U/mg protein. For CAT activity assay one unit was defined as the amount of enzyme that will cause the formation of 1.0 nmol of formaldehyde per minute at 25°C. CAT activity was expressed as nmol of formaldehyde/mg protein.min.

### Live imaging of superoxide formation

Retinal explants were placed photoreceptor cell side down in glass-bottom culture plate (μ-dish 35 mm low, Ibidi cell in focus, Madrid, Spain) and incubated in Kreb´s solution (118 mM NaCl; 4.7 mM KCl; 1.2 mM MgSO_4_; 1.5 mM MgSO_4_, 1.5 mM CaCl_2_, 25 mM NaHCO_3_, 1.2 mM KH_2_PO_4_, 11 mM Glucose at pH 7,4) with 10 μM dihydroethidium (DHE) (Sigma-Aldrich, Madrid, Spain) and 1,5 μM Hoescht 33342 (Sigma-Aldrich, Madrid, Spain) for 30 minutes at 37°C in a humidified incubator. Fluorescence was visualized with a confocal microscope at 63x magnification (Leica TCS SP5 Confocal microscope, Leica Microsystems CMS GmbH, Mannheim, Germany). Leica LAS AF was used as microscope imaging software (Leica Microsystems CMS GmbH, Mannheim, Germany). Serial optical sections were recorded every 0.5 μm along 20 μm. Images were taken with standardized intensity settings on filter channels to ensure no bleed through of immunofluorescence between the channels.

ImageJ software was used to quantify the intensity of DHE staining at each timepoint. DHE staining was represented as the ratio between the number of nuclei DHE positive and number of total nuclei (Hoechst staining).

### Statistical analyses

Data were summarized using mean (standard deviation) and median (1^st^– 3^rd^ quartile) in the case of continuous variables and by absolute and relative frequencies in the case of categorical variables. For parametric data, ANOVA followed by Newman-Keuls post hoc test was used. Effects of Zaprinast, KT5823 and hypoxia on the different response variables were assessed using mixed effects linear regression models. An interaction between Zaprinast and KT5823 was added to the models to assess non-additive effects between both treatments. P values lower than 0.05 were considered statistically significant. All analyses were performed using R (version 3.2.2, Foundation for Statistical Computing, Vienna, Austria).

## Results

### Mild hypoxia induced tissue hypoxia increasing lactate/pyruvate ratio and gene expression of HIF-1α targets

Porcine retinal explants were cultured for 24 h under mild hypoxic conditions (5% O_2_). In order to ascertain whether this condition induced tissue hypoxia we measured lactate/pyruvate (Lac/Pyr) ratio [40] and expression of a subset of hypoxia inducible factor- 1 alpha (HIF-1α) signature target genes: EPO, VEGF, iNOS and ADM[41] in retinal explants. We determined that the mean ± SEM of Lac/Pyr ratio was higher in hypoxic retinal explants [148 ± 14] than in normoxic retinal explants [86 ± 26]. Concerning gene expression of HIF-1α signature target genes, we observed an increase in VEGF (p < 0.05), ADM (p < 0.01), and EPO (p < 0.01) genes, but no change in iNOS gene (Table 1). Thus these results indicated that 5% O_2_ produced tissue hypoxia in these explants.

**Table 1:** Gene expression of a subset of HIF-1α signature target genes reflecting tissue hypoxia in porcine retinal explants exposed to mild hypoxia.

Gene expression	Treatment (% of Oxygen)	mean (SD)	median (1^st^, 3^rd^ Q)
**EPO**	***21%***	1.0 (0.03)	1.0 (0.97, 1.03)
	***5%***	1.9 (0.6)	1.8 (1.3, 2.4)
**VEGF**	***21%***	1.0 (0.1)	1.0 (0.9, 1.1)
	***5%***	1.7 (0.03)	1.8 (0.9, 2.2)
**INOS**	***21%***	1.0 (0.03)	1.0 (0.97, 1.03)
	***5%***	1.3 (0.6)	1.2 (0.9, 1.5)
**ADM**	***21%***	1.0 (0.03)	1.0 (0.97, 1.03)
	***5%***	1.6 (0.3)	1.6 (1.2, 1.9)

### Hypoxia dramatically decreased cGMP in porcine retinal explant

**Fig 1:**
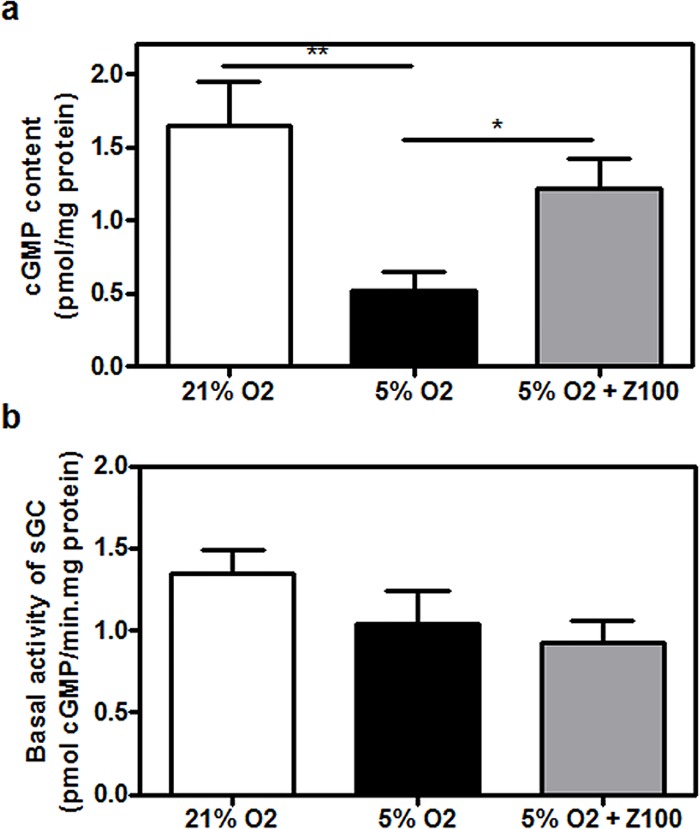
Hypoxia dramatically decreased cGMP in cultured porcine retina. Retinal explants were incubated under normoxia (21% of oxygen) or mild hypoxia conditions (5% of oxygen) for 24h with dimethyl sulfoxide (DMSO) or Zaprinast as described in Material and Methods. cGMP content (**a**) and basal sGC activity (**b**) in retinal homogenates. Values are the mean ± SEM of six different cultures. Values that are significantly different are indicated by asterisks * P < 0.05; ***P < 0.001 (linear regression models of mixed effects). Z100: 100 nM Zaprinast; sGC: soluble guanylate cyclase.

Porcine retinal explants were cultured for 24 h under mild hypoxic conditions (5% O_2_) in the presence or absence of 100 nM Zaprinast which raises intracellular cGMP levels in a concentration-dependent manner in normoxic explants [38]. At this working concentration Zaprinast should substantially inhibit PDE6 (Ki = 30–32 nM) (see Material and Methods section). We analyzed whether Zaprinast raised cGMP in hypoxic retinal explants by measuring cGMP content (Fig 1A). Surprisingly cGMP content significantly decreased in untreated hypoxic retinal explants (0.5 ± 0.1 pmol/mg protein, p<0.001) compared to normoxic retinal explants (1.7 ± 0.3 pmol/mg protein). Zaprinast, although it did not completely restore control values, increased cGMP content to 1.2 ± 0.2 pmol/mg protein (p<0.05) in hypoxic retinal explants.

The reduced cGMP content observed in hypoxic explants could result from hypoxia regulating redox processes and in turn, directly influencing sGC activity, the enzyme that catalyzes the synthesis of cGMP [42]. To further explore this possibility we measured *in vitro* sGC activity in homogenates of retinal explants (Fig 1B). We found that hypoxia similarly decreased sGC activity in hypoxic untreated explants (0.9 ± 0.1 pmol/mg protein.min, p<0.01) and Zaprinast-treated explants (0.9 ± 0.1 pmol/mg protein.min (p<0.05).

### PDE inhibition prevented mild hypoxia-induced cell death through PKG activation

**Fig 2:**
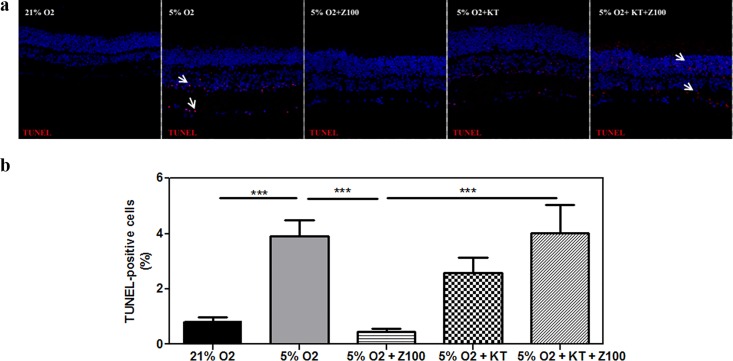
PDE inhibition prevented hypoxia-induced cell death through PKG activation in cultured porcine retina. Retinal explants were incubated under normoxia (21% of oxygen) or mild hypoxia conditions (5% of oxygen) for 24h with dimethyl sulfoxide (DMSO), Zaprinast and KT5823 alone or combined with Zaprinast as described in Material and Methods. Confocal laser scanning micrographs of retinal sections showing TUNEL-stained sections visualizing apoptotic cells (pink) in DAPI-counterstained retinal sections. Scale bar: 50 μm. **(a)**. Bar graphs showing the quantification of TUNEL **(b).** Values are the mean ± SEM of six different cultures. Values that are significantly different are indicated by asterisks ***P < 0.001 (linear regression models of mixed effects). Z100: 100 nM Zaprinast; KT: 1 μM KT5823; TUNEL, terminal deoxynucleotidil transferase dUTP nick-end labeling.

To test whether hypoxic condition induced cell death in porcine retinal explants we performed TUNEL staining. As shown in Table 2 and Fig 2 mild hypoxia increased TUNEL-positive cells respect to total cells (3.9 ± 0.6% of TUNEL-positive cells respect to total cells, p < 0.001) in untreated explants compared to normoxic retinal explants (0.8 ± 0.1% of TUNEL-positive cells respect to total cells) through all the nuclear layers of the retina.

**Table 2:** PDE inhibition reduced mild hypoxia-induced cell death in porcine retinal explants.

	Treatment	ONL	INL	GCL
		*mean (SD)*	*mean (SD)*	*mean (SD)*
		*median (1*^*st*^, *3*^*rd*^ *Q)*	*median (1*^*st*^, *3*^*rd*^ *Q)*	*median (1*^*st*^, *3*^*rd*^ *Q)*
	***21%***	0.04 (0.08)	0.45 (0.51)	0.25 (0.26)
		0 (0, 0)	0.3 (0, 0.66)	0.17 (0, 0.46)
	***5%***	1.08 (2.13)	1.54 (1.46)	0.9 (1.14)
		0.36 (0.1, 1.04)	1.05 (0.47, 2.1)	0.52 (0, 1.2)
**TUNEL-positive cells (%)**	***5%+Z100***	0.21 (0.37)	0.15 (0.37)	0.08 (0.18)
		0 (0, 0.26)	0 (0, 0.11)	0 (0, 0.07)
	***5%+KT***	0.5 (0.94)	2.54 (2.31)	0.92 (0.98)
		0.13 (0, 0.45)	2.07 (0.95, 3.73)	0.9 (0.15, 1.1)
	***5% +KT+Z100***	0.6 (0.58)	2.52 (2.43)	0.9 (1.06)
		0.66 (0.04, 0.72)	1.4 (0.88, 4.52)	0.58 (0, 1.51)
	***21%***	0.37 (0.60)	0.25 (0.65)	0.21 (0.31)
		0.3 (0, 1.45)	0 (0, 1.35)	0.2 (0, 0.35)
	***5%***	1.12 (1.27)	2.5 (1.97)	1.19 (0.94)
		0.65 (0.3, 1.3)	2.35 (1.43, 3.25)	1.05 (0.68, 1.5)
**PAR-positive cells (%)**	***5%+Z100***	0.55 (1.04)	0.84 (1.48)	0.28 (0.41)
		0.2 (0, 0.5)	0.3 (0, 0.5)	0 (0, 0.3)
	***5%+KT***	1.34 (1.12)	0.9 (1.20)	0.48 (0.49)
		1.1 (0.4, 2.4)	0.3 (0.2, 0.8)	0.5 (0, 0.6)
	***5%+KT+Z100***	2.81 (0.94)	0.23 (0.35)	0.45 (0.38)
		2.85 (2.4, 2.98)	0.1 (0, 0.2)	0.5 (0.23, 0.5)
	***21%***	0 (0)	0.19 (0.38)	0.4 (0.55)
		0 (0, 0)	0 (0, 0.26)	0 (0, 0.69)
	***5%***	0.11 (0.15)	2.67 (1.44)	1.28 (0.44)
		0 (0, 0.27)	3.19 (1.38, 3.53)	1.35 (1, 1.54)
**CP3-positive cells (%)**	***5%+Z100***	0 (0)	0.67 (0.28)	0.85 (0.36)
		0 (0, 0)	0.74 (0.43, 0.81)	0.85 (0.59, 1.1)
	***5%+KT***	0.51 (0.66)	2.55 (1.58)	0.84 (0.57)
		0.41 (0, 1.05)	2.46 (1.59, 3.39)	1.07 (0.34, 1.15)
	***5%+KT+Z100***	0 (0)	3.95 (2.39)	1.36 (0.6)
		0 (0, 0)	3.6 (2.22, 6.32)	1.44 (1.12, 1.86)

Hypoxia-induced cell death through all the nuclear layers. However, the highest increase of cell death respect to normoxia condition was detected at ONL (from 0.04% to 1.08%, 25-fold) (Table 2). PDE inhibition drastically reduced the number of hypoxia-induced TUNEL-positive cells from 3.9 ± 0.6% to 0.5 ± 0.1% (p < 0.001) through all the nuclear layers (Fig 2B). cGMP accumulation, therefore, presents a neuroprotective effect against hypoxia in porcine retinal explants. To assess whether cGMP mediated their protective effects via the cGMP-PKG pathway we exposed hypoxic explants to 1 μM KT5823, a potent, selective and permeable PKG inhibitor (Ki = 0.05–0.1 μM) that can also inhibit PKC and PKA at higher concentrations (Ki = 4 μM and Ki >10 μM, respectively) (see Material and Methods section). **A**s shown in Fig 2, the protective effect of Zaprinast disappeared when hypoxic explants were treated with KT5823 (4.0 ± 1.0% of TUNEL-positive cells). The effect of PKG inhibition was stronger in the INL (2.5 ± 0.7% of TUNEL-positive cells) than in ONL (0.6 ± 0.2% of TUNEL-positive cells) or GCL (0.9 ± 0.3% of TUNEL-positive cells) in Zaprinast-treated retinal explants (Table 2).

These results indicate that mild hypoxia induces cell death through all retina layers and cGMP accumulation can prevent cell death through PKG activation.

### PDE inhibition prevented caspase-3 and PARP activation under mild hypoxia

During brain or retinal hypoxia/ischemia several cell death mechanisms have been postulated: apoptotic, necrotic or autophagic. We assessed whether hypoxia induced cell death via caspase-dependent or -independent mechanisms such as PARP activation.

**Fig 3:**
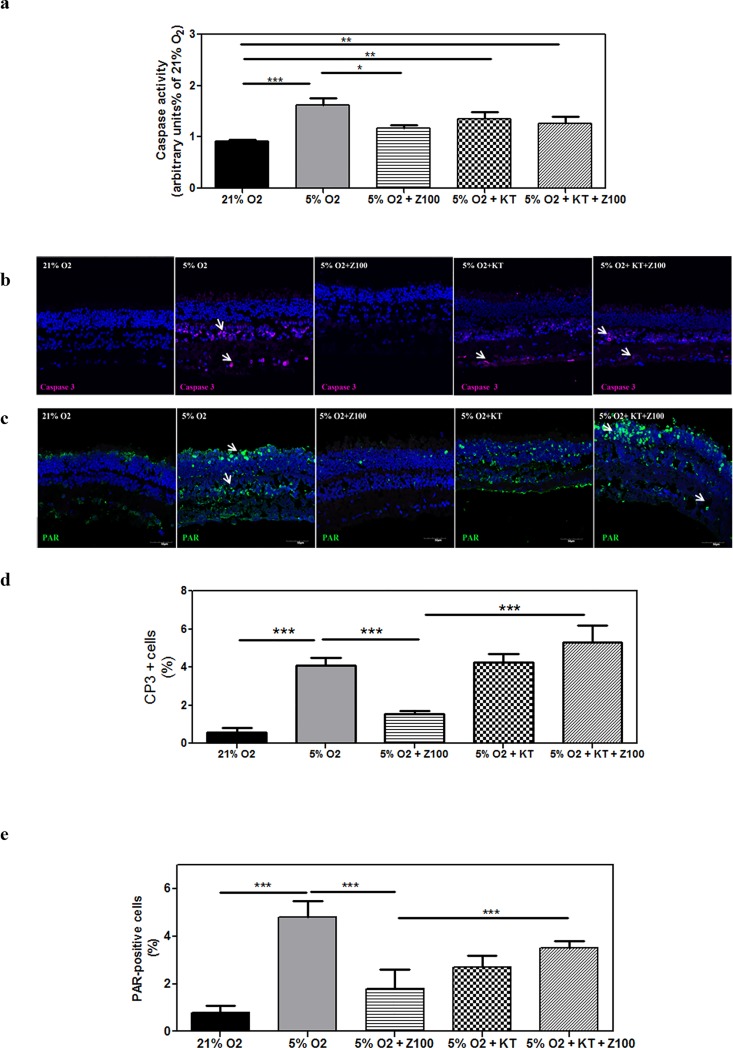
PDE inhibition prevented *caspase-3* and PARP activation under hypoxia in cultured porcine retina. Retinal explants were incubated under normoxia (21% of oxygen) or mild hypoxia conditions (5% of oxygen) for 24h with dimethyl sulfoxide (DMSO), Zaprinast and KT5823 alone or combined with Zaprinast as described in Material and Methods. *Caspase*-3 activity using the substrate DEVD-pNA in retinal homogenates **(a)**. Confocal laser scanning micrographs of retinal sections showing cleaved *caspase*-3 positive cells (pink) **(b)** and PAR accumulation (green) **(c)** in DAPI-counterstained retinal sections. Scale bar: 50 μm. Bar graphs showing the quantification of cleaved *caspase-3* (**d**) and PAR accumulation. **(e)** Values are the mean ± SEM of six different cultures. Values that are significantly different are indicated by asterisks ***P < 0.001 (mixed effects linear regression models). Z100: 100 nM Zaprinast; KT: 1 μM KT5823; CP3: cleaved caspase 3; PAR: polyADP ribose polymers.

After 24 h of mild hypoxia, *caspase*-3 activity increased two-fold (1.7 ± 0.1 a.u/mg protein) with respect to normoxic condition in untreated explants (0.9 ± 0.04 a.u/mg protein, p < 0.001). Zaprinast significantly reduced *caspase*-3 activity (1.2 ± 0.04 a.u/mg protein, p<0.05) in porcine retinal explants exposed to hypoxia (Fig 3A). Hypoxic explants treated with Zaprinast plus KT5823 present similar values of *caspase*-3 activity (1.4 ± 0.07 a.u/mg protein) that untreated explants suggesting that PKG was involved in the Zaprinast-induced *caspase*-3 inhibition (Fig 3A). Immunostaining of cleaved *caspase-3* corroborated that hypoxia increased the percentage of *caspase-3-* positive cells respect to total cells (4.1 ± 0.4% CP3-positive cells respect to total cells, p < 0.001) compared to normoxic retinal explants (0.6 ± 0.2% CP3-positive cells respect to total cells) (Fig 3B and 3D). In particular, cleaved *caspase*-3 significantly increased in the INL (p < 0.001) (Table 2). Zaprinast reduced the percentage of caspase-3-positive cells (1.5 ± 0.1% CP3-positive cells, p < 0.001). When we analyzed the effect of KT5823 on Zaprinast-treated explants we found that total cleaved *caspase*-3-positive cells increased (5.3 ± 0.9% CP3-positive cells, p < 0.001) (Fig 3D). The analysis of each layer indicated that this increase mainly occurred in the INL (p < 0.001), suggesting that cGMP-PKG pathway could mediate *caspase*-3 inhibition only in this layer (Table 2).

We also analyzed the accumulation of poly (ADP-ribose) polymers (PAR) as an indirect measure of PARP activation which has been involved in cell death under hypoxic/ischemic conditions (Fig 3C and 3E). Immunostaining of PAR revealed a significant accumulation of these polymers in cell nuclei of all retinal layers (4.8 ± 0.7% PAR-positive cells respect to total cells, p < 0.001) compared to normoxic retinal explants (0.8 ± 0.3% PAR-positive cells respect to total cells). Zaprinast reduced the percentage of PAR-positive cells (1.8 ± 0.8% PAR-positive cells, p < 0.001). When we analyzed the effect of KT5823 on Zaprinast-treated explants we found that PAR-positive cells significantly increased (3.5 ± 0.3% PAR-positive cells, p < 0.001) (Fig 3E). However, the analysis of each nuclear layer revealed that PKG activation could be responsible for PARP inhibition only in the ONL (p < 0.001) but not in the INL and GCL (Table 2).

### PDE inhibition ameliorated the effect of mild hypoxia on antioxidant response and the release of superoxide free radical

**Fig 4:**
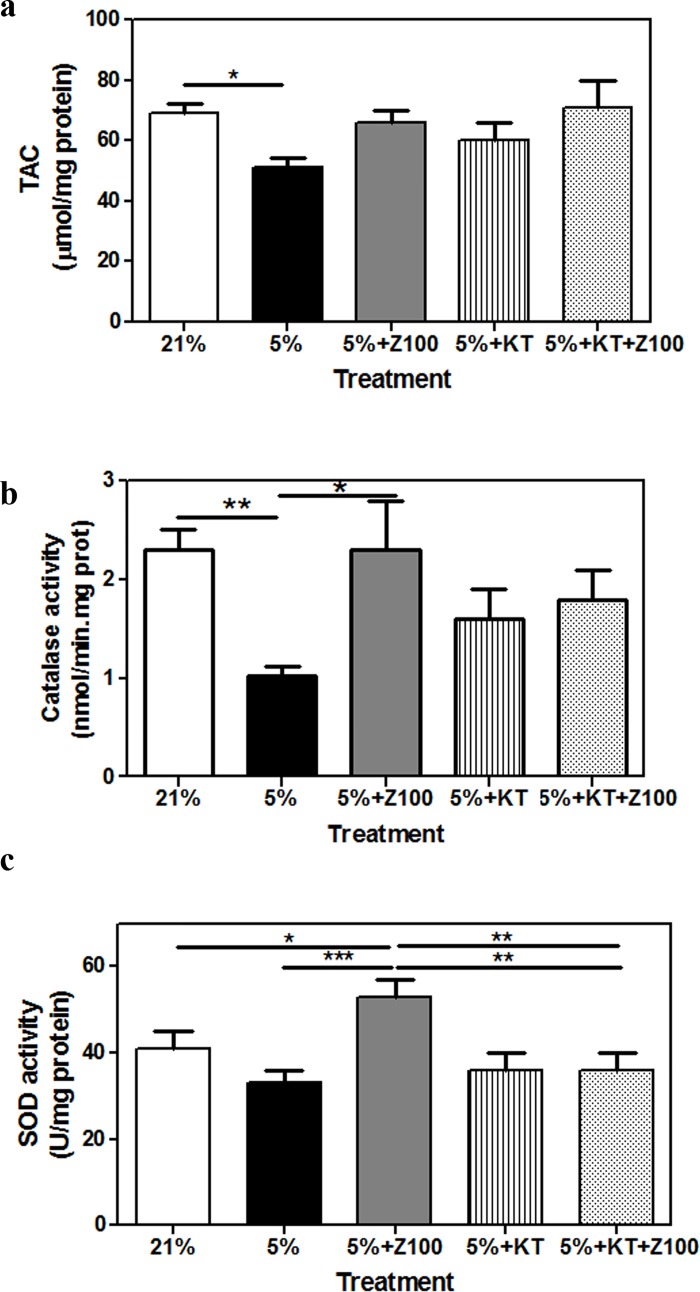
PDE inhibition ameliorated the effects of hypoxia on antioxidant response in cultured porcine retina. Retinal explants were incubated under normoxia (21% of oxygen) or mild hypoxia conditions (5% of oxygen) for 24h with dimethyl sulfoxide (DMSO), Zaprinast and KT5823 alone or combined with Zaprinast as described in Material and Methods. Total antioxidant capacity (**a**), superoxide dismutase (**b**) and catalase (**c**) activities. Values are the mean ± SEM of seven different cultures. Values that are significantly different are indicated by asterisks *P < 0.05, **P < 0.01, ***P < 0.001 (linear regression models of mixed effects). Z100: 100 nM Zaprinast; KT: 1 μM KT5823; SOD: superoxide dismutase.

We analyzed whether mild hypoxia induced changes on antioxidant response and the content of oxidative markers in porcine retinal explants and whether Zaprinast had any beneficial effect. To evaluate antioxidant response we measured total antioxidant capacity (TAC) and the enzymatic activity of superoxide dismutase (SOD) and catalase (CAT) which play a major role in the first line of antioxidant defence system. As shown in Fig 4, mild hypoxia reduced antioxidant response. Hypoxia significantly decreased TAC levels and CAT activity (p < 0.05) respect to normoxic condition (Fig 4A and 4B) and showed a tendency to reduce SOD activity (Fig 4C). Zaprinast ameliorated antioxidant response in porcine retinal explants exposed to hypoxia (Fig 4). Similar values were found for TAC and CAT activity in hypoxic explants treated with Zaprinast or with Zaprinast plus KT5823 suggesting that the putative neuroprotective effect of cGMP was PKG-independent for these markers of antioxidant response. However, the effect of Zaprinast on SOD activity disappeared after inhibition of PKG (p < 0.01).

**Fig 5:**
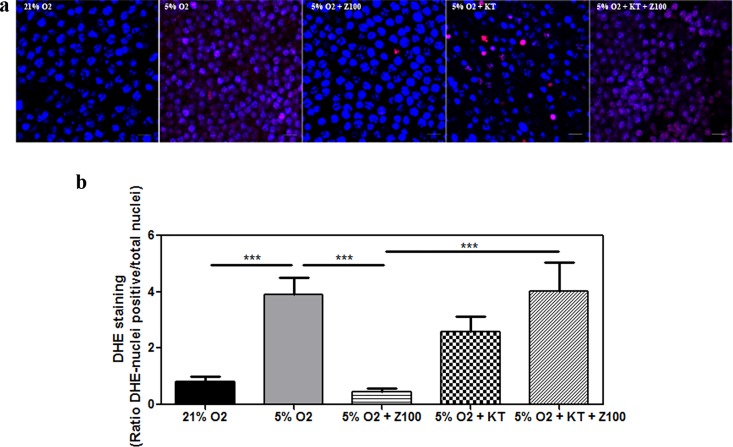
PDE inhibition ameliorated the effects of hypoxia on superoxide release in cultured porcine retinas. Retinal explants were incubated under normoxia (21% of oxygen) or mild hypoxia conditions (5% of oxygen) for 24h with dimethyl sulfoxide (DMSO), Zaprinast and KT5823 alone or combined. Superoxide radical generation was assessed with the oxidative fluorescent dye dihydroethidium (DHE) in the outer nuclear layer (photoreceptors) as described in Material and Methods. Confocal laser scanning micrographs showing DHE imaging of superoxide accumulation (red). Scale bar: 10 μm (**a**) in Hoescht 33342-counterstained of live retinal explants, Bar graphs showing the quantification of superoxide (**b)**. Values are the mean ± SEM of five different cultures. Values that are significantly different are indicated by asterisks ***P < 0.001 (linear regression models of mixed effects). Z100: 100 nM Zaprinast; KT: 1 μM KT5823; TAC, total antioxidant capacity.

To further analyze whether the apparent reduction of SOD activity induced by hypoxia could correlate with high production of superoxide anion, we performed live imaging of this free radical (Fig 5). We incubated live retinal explants (photoreceptor cells side down) with dihydroethidium (DHE) that has the ability to freely permeate cell membranes and reacts with superoxide anions forming a red fluorescent product (ethidium) which intercalates with DNA [43, 44]. Explants were stained with Hoechst dye that stained only active cell nuclei. Hypoxic explants showed an intense DHE staining (3.9 ± 0.6 a.u, p < 0.001) compared to normoxic explants (0.8 ± 0.2 a.u) that disappeared after Zaprinast treatment (0.5 ± 0.1 a.u, p < 0.001). Hypoxic explants treated with Zaprinast plus KT5823 showed similar overproduction of the superoxide radical (4.0 ± 0.1 a.u) to untreated hypoxic explants (Fig 5A and 5B). These results support the idea that SOD activity and the consequent production of superoxide radical are controlled by the cGMP-PKG pathway under hypoxic conditions.

## Discussion

Retinal hypoxia and oxidative stress have been implicated in the pathophysiology of several retinopathies such as age related macular degeneration (AMD), retinopathy of prematurity, central retinal vein occlusion (CRVO), diabetic retinopathy, glaucoma or branch retinal vein occlusion (BRVO) [4, 12, 45–47]. During ischemic retinal injury failure of energy metabolism and excessive stimulation of glutamatergic neurotransmission lead to neuronal depolarization, increase in intracellular calcium, accumulation of free radicals and subsequent cell death. An increasing number of treatments are focused on blocking any step of the ischemic cascade to ameliorate the deleterious effects of retinal ischemia. However, current therapies do not significantly improve vision impairment in patients. Furthermore, neuroprotection-based treatment strategies for retinal ischemia have so far been disappointing.

In the current study we assessed whether mild hypoxia (5% O_2_) induced retinal degeneration in an *ex vivo* porcine retina explant model. This *ex vivo* model has some limitations as previously shown under normoxic conditions [38] for instance culturing time and retinal detachment increase cell death beyond 24 h in culture; however our model showed clear signs of hypoxia, since we observed that these explants had high Lac/Pyr ratio (i.e. a widely recognized sign of hypoxia) and even we detected upregulation of a subset of HIF-1α target genes.

We firstly assessed whether mild hypoxia induced retinal degeneration and oxidative stress in an *ex vivo* porcine retina explants. Then we evaluated whether the accumulation of the second messenger cGMP induced by Zaprinast was able to protect against the hypoxia-induced retinal degeneration.

In neural tissue PDEs are considered therapeutic targets because they are involved in many basic functions such as synaptic plasticity, homeostasis, regulation of the glial inflammatory response, and a number of basic behaviors including cognition or anxiety. PDEs are key regulators of the second messenger cyclic nucleotides (cGMP and cAMP). Although the role of the cGMP-degrading PDE remains unclear, evidence suggests that their inhibition may be a useful therapeutic intervention after brain injury [48–51].In particular, inhibitors of PDE5 or PDE6 have been used to prevent cell death under hypoxic conditions [52]. For instance, tadalafil reduced cell death, caspase activity and increased PKG activity in an in vitro model of ischemia/reperfusion [53] and yonkenafil reduced cell death in a model of acute experimental stroke [54].

Drugs that selectively and potently target PDE5 (e.g. Zaprinast, Sildenafil, Vardenafil or Tadalafil) are also excellent PDE6 inhibitors because of the structural, biochemical and pharmacological similarities of these both PDEs. As far as we know there are no specific PDE6 inhibitors but Zaprinast, when used in the appropriated concentration, is considered a “PDE6 selective” inhibitor. It is the only drug that inhibits PDE6 more potently than PDE5 [39].

We checked the content of cGMP and the effect of Zaprinast under hypoxia in this ex *vivo* model. Hypoxia promoted a drastic reduction of cGMP content that in turn was increased in presence of Zaprinast. Redox processes that occur during hypoxia could influence the cGMP production by sGC, the removal of cGMP by PDEs, and protein phosphorylation processes regulated by PKG. Since the activity of sGC is directly regulated by multiple reactive oxygen species (ROS), thiol and heme redox reactions [55], we evaluated whether reduced cGMP content found in hypoxia was a consequence of reduced sGC activity. *In vitro* measurement of sGC activity suggested that hypoxia could reduce cGMP content through inhibition of sGC activity.

cGMP metabolism is essential for photoreceptor physiology. In the light, rhodopsin catalyzes the activation of the G protein transducin which activates the PDE6 to hydrolyze cGMP. The decrease of cGMP concentration leads to the closure of the cGMP-gated channels reducing sodium influx and hyperpolarizing the photoreceptor. The voltage-gated calcium channels close and glutamate release decreases at the synaptic terminal. This signal is processed by the inner retina before being transmitted to the brain. In the dark, cGMP concentration is high and cGMP-gated sodium channels are opened, the photoreceptor depolarizes, voltage-gated calcium channels open leading to glutamate release. In this study we observed that hypoxia reduced cGMP concentration suggesting that the phototransduction cascade did not work properly. Electrophysiological tests support this idea. For instance it has been shown that hypoxia or strong illumination can produce lack of the ERG a-wave (hyperpolarization of the photoreceptors) or change its response [56, 57]. This type of waves can be related to changes on the phototransduction cascade by assessing changes in its slope. PDE6 inhibition partly restored cGMP concentration and this could improve the phototransduction cascade in hypoxic explants. Retinal hypoxia/ischemia affects most neuronal cells types (photoreceptors, bipolar and ganglion cells) but ganglion cells are especially vulnerable [58]. Under our experimental conditions, we corroborated that mild hypoxia induced retinal degeneration through the whole porcine retinas by increasing TUNEL-positive cells. Inhibition of PDE6 by Zaprinast drastically reduced cell death through the retina. Our results corroborate evidence supporting a neuroprotective role of the sGC-cGMP pathway for neuronal cells [59] and other cells against hypoxia [53]. Retina is a well-organized structure formed by cell layer and microcircuits that work together to encode visual information. Cell death of a specific retinal cell type or types can lead to changes in the retinal organization and contribute to a secondary degeneration [60].

It could be possible that inhibition of PDE6 would increase cGMP content in photoreceptors avoiding their degeneration, contributing to preserve synaptic connections, and eventually promoting the survival of other retinal cells (e.g bipolar and ganglion cells).

The use of a PKG inhibitor, KT5823, cancelled the protective effect of Zaprinast increasing the number of TUNEL-positive cells suggesting cGMP-PKG pathway is involved in cell survival under retinal hypoxia. The primary target of cGMP is PKG, a serine-threonine kinase (Iα and Iβ isoforms). When bound to cGMP, both isoforms of PKG phosphorylate serine threonine residues in a broad variety of target proteins kinase as AKT, phosphatidylinositol 3' kinase (PI3K) or cAMP-responsive element binding (CREB), that are implicated in regulation of cell functions such as survival, the calcium signaling pathway, transcription, platelet activation, cell growth or differentiation [61]. During hypoxia PKGs seem to be the main effectors of cGMP especially for endothelial dysfunction [62].

As previously described by other authors, the retinal degeneration induced by hypoxia or ischemia-reperfusion can be via caspase-dependent or independent mechanisms such as the activation of calpains (by increased intracellular calcium) [63, 64] or excessive activation of the nuclear PARP (necroptosis or apoptosis). Under our experimental conditions, we observed that mild hypoxia promoted cell death through *caspase*-3 activation in the inner retina (INL and GCL) and PARP activation through the whole retina. cGMP accumulation induced by Zaprinast prevented cell death and PARP activation at all nuclear layers and, to a lesser extent, *caspase*-3 activation in the inner retina. However, the involvement of PKG on these protective effects depends on the analyzed cell layer. In the outer retina (ONL), KT5823 partially reversed the neuroprotective effect of Zaprinast and the PARP inhibition suggesting that the cGMP-PKG pathway was involved in PARP inhibition and to a lesser extent cell survival. Hypoxia did not induce *caspase*-3 activation in the ONL. In the inner retina, KT5823 reversed the neuroprotective effect of Zaprinast and *caspase*-3 inhibition suggesting that the cGMP-PKG pathway was involved in cell survival through the inhibition of *caspase*-dependent mechanisms. However, PARP inhibition induced by Zaprinast was not mediated through the cGMP-PKG pathway in the inner retina. These results, therefore, suggest that cell death induced by hypoxia is mediated by different cGMP-dependent mechanisms in different cell layers. Pathologic susceptibilities are different in outer and inner neurons under hypoxic damage. Evidence suggests that cell death mechanisms are mainly mediated by excitotoxicity (high glutamate levels and overstimulation of ionotropic receptors (NMDA or AMPA) on the inner retina after ischemic or hypoxic insults [65, 66]. However, other mechanisms could be involved in the outer retina. For instance the reduced cGMP found during hypoxia could compromise photoreceptor functional response dynamics (phototransduction) and lead it to cell death through PARP activation. Overactivation of PARP in photoreceptors has been also observed when cGMP is high as happens in retinitis pigmentosa, an inherited retinal degenerations [67, 68]. In retinitis pigmentosa, this increased cGMP is responsible for upregulation of PARP activity through PKG [69, 70]. In any case, experimental conditions (*in vivo* or *in vitro* models), duration of the hypoxic or ischemic insult, type of *in vivo* model, age (neonatal or adult retina), etc can trigger different cell death mechanisms.

Although cGMP effects are frequently mediated by activation of a PKG, cGMP can also act to regulate other targets, including cyclic nucleotide-gated ion (CNG) channels and phosphodiesterases [71–73]. Our data indicate that other downstream effectors of cGMP should contribute to the downregulation of PARP activity induced by Zaprinast in the INL (mainly bipolar cells) and GCL (ganglion cells) and to the cell survival in the ONL (photoreceptor cells). Further studies are needed to clarify this point.

As previously commented, many studies indicate that oxidative stress plays a crucial role in the pathogenesis of several pathologies associated to hypoxia including retinal ischemia/hypoxia [74, 75]. Oxidative stress occurs when the production of ROS or RNS exceed the endogenous antioxidant response mediated by the enzymes CAT or SOD, vitamins or carotenoids, among others. ROS triggers several signaling pathways (e.g. JNK/p38 MAPK, PARP, JAK/STAT, NFkB, etc), and can cause macromolecular damage, leading to cell death. Antioxidants can inhibit or prevent the oxidative processes protecting retinal cells from ischemic damage. In several ischemic retinopathies overproduction of ROS and RNS and reduced antioxidant response were found [76–78]. Several pharmacological approaches based on antioxidant therapies have been used to reduce free radical and increase antioxidant defense in ischemic/hypoxic retinopathies [5, 79, 80]. In the current study, we found that hypoxia reduced antioxidant response (TAC and, CAT activity), and increased the level of the superoxide radical. PDE inhibition significantly ameliorated antioxidant response and reduced superoxide radical formation in the ONL. Our results suggest that cGMP-PKG pathway mediated these protective effects by increasing SOD activity and reducing superoxide radical formation as observed in other ischemic situations [77].

We propose that retinal hypoxia could down-regulate PKG activity by two different mechanisms: i) the reduced cGMP content found in hypoxic explants could lead to reduced PKG activity or ii) the presence of ROS and RNS could induce posttranslational modifications (e.g nitration) reducing its activity [81]. As mentioned above, hypoxic retinal explants showed elevated content of superoxide radical. This radical can react with nitric oxide to form peroxynitrites (ONOO^−^), a potent and versatile oxidant that can nitrate tyrosine residues of PKG and decrease their activity [81]. Accumulation of cGMP induced by Zaprinast, therefore, could restore PKG activity by increasing cGMP content or reducing superoxide radical formation, having protective effects although the distinct cellular downstream targets for these effects remained unknown. The NO-sGC-cGMP-PKG signaling pathway also participates in the survival or death processes in several neuronal diseases e.g. amyotrophic lateral sclerosis, Alzheimer's disease, HIV dementia and Parkinson's disease, retinitis pigmentosa, etc [70, 82]. For instance, it has been described that PKG prevented from cell death through P38MAPK inhibition in ischemic cardiomyocytes or through phosphorylation of CREB in serum deprived R28 cells [83, 84]. However, under other pathological situations, excessive activation of PKG is responsible for the death of neuronal and non-neuronal cells [85–87].

In summary, the present work shows that the use of the PDE inhibitor, Zaprinast, prevented hypoxia-induced cell death and improved redox status in an *ex vivo* model of porcine retinal explants by different cGMP-dependent mechanisms in different cell layers. The inhibition of PDE, therefore, could be useful for reducing retinal degeneration under hypoxic/ischemic conditions. Besides we believe that this *ex vivo* model of mild hypoxia could be useful for studying some aspects related to the retinal degeneration induced by hypoxia and testing potential therapies.
